# Supplementary material for: Sustained Consumption of a Decaffeinated Green Coffee Nutraceutical Has Limited Effects on Phenolic Metabolism and Bioavailability in Overweight/Obese Subjects
Source: Nutrients. 2022 Jun 13;14(12):2445. doi: 10.3390/nu14122445 (PMC9230201; doi:10.3390/nu14122445)
Supplement: Supplementary file 1 [file nutrients-14-02445-s001.zip › nutrients-1754691-supplementary.pdf]

LC-Q-ToF ANALYSIS OF BIOLOGICAL SAMPLES

Plasma (30 µL), urine (5 µL) and fecal extracts (5 µL) were filtered through 0.45 µm cellulose-acetate membrane filters and injected into an Agilent 1200 series LC system coupled to an Agilent 6530A Accurate-Mass Quadrupole Time-Of-Flight (Q-ToF) with ESI-Jet Stream Technology (Agilent Technologies). A reverse-phase Ascentis Express C18 (15 cm x 3 mm, 2.7 µm) column (Sigma-Aldrich Quimica, Madrid), preceded by a Supelco 55215-U guard column (3 mm x 5 mm, 2.7 µm), was used for separation. Mobile phase A was 0.1% formic acid in Milli-Q water, and mobile phase B was acetonitrile containing 0.1% formic acid at a 0.3 mL/min flow rate. The gradient started with 10% B at 0 min, 30% B at 10 min, 40% B at 15 min, reaching 50% B at 20 min. The starting gradient was then re-established in 2 min and maintained for 8 min to re-equilibrate the column. Q-ToF acquisition conditions were as follows: drying gas flow (nitrogen, purity > 99.9%) and temperature were 10 L/min and 325°C, respectively; sheath gas flow and temperature were 6 L/min and 250°C, respectively; nebulizer pressure was 25 psi; cap voltage was 3500 V, and nozzle voltage was 500 V. Mass range selected was from 100 up to 970 *m/z* in negative mode and fragmentor voltage of 150 V. Data were processed in a Mass Hunter Workstation Software.

For metabolite identification and quantitation, calibration curves of available standards were freshly prepared in a pool of plasma, urine and fecal water, respectively, to account for potential matrix effects or losses during sample processing. Phase II metabolites for which there were no commercially available authentic standards were tentatively quantified using the calibration curves of their corresponding phenolic precursors as follows: monohydroxycinnamoyl derivatives were quantified using 5-caffeoylquinic acid as standard; free hydroxycinnamic acids and phase II metabolites of the corresponding parent compounds were quantified with the respective calibration curves of 3′,4′-dihydroxycinnamic acid, 4′-hydroxy-3′-methoxycinnamic acid, 4′-hydroxycinnamic acid, 3-(3′,4′-dihydroxyphenyl)propanoic acid, 3-(4′-hydroxy-3′-methoxyphenyl)propanoic acid, and 3-(4′-hydroxyphenyl)propanoic acid.

All the standard curves showed a linear response from 5 to 5000 nM as checked by linear regression analysis. Inter- and intra-day precision were considered acceptable (calculated as the coefficient of variation, ranging from 2.5% to 8.5%). Percentage of recovery, and limits of detection (LOD) and quantification (LOQ) are shown bellow.

	**Plasma**	**Urine**	**Feces**
**Recovery (%)**	94-102%	95-99%	96-101%
**LOD (nM)**	1.5-4	3-20	0.2-41
**LOQ (nM)**	3-10	30-70	0.8-136

INTER-INDIVIDUAL VARIABILITY OF PHENOLIC METABOLITES IN PLASMA, URINE AND FECES AT BASELINE AND AFTER 8-WEEK CONSUMPTION OF THE GREEN COFFEE PHENOLIC EXTRACT (GCPE) NUTRACEUTICAL

To better explore the high inter-individual variability in the excretion profiles of phenolic metabolites in urine, feces and also in plasma kinetics, an unsupervised principal components analysis (PCA) was carried out for each biofluid:

PLASMA: In this matrix, three principal components (PCs) explained 67.7% of the total variability:

- The first PC described the 48.2% of the observed variation and was positively correlated with a high presence of dihydroferulic acid (DHFA), DHFA-4-glucuronide, DHFA-4-sulfate, DHFA-3-glucuronide, dihydro*iso*ferulic acid (DHiFA), dihydrocaffeic acid (DHCA), as well as 4-hydroxyhippuric acid (circled in green); and inversely correlated with dihydrodimethoxycinnamic acid (DHDMCA), 4-hydroxybenzoic acid and 4-hydroxy-3-methoxybenzoic acid.
- PC2 explained 11.2% of variability and was positively loaded by 5-feruloyl quinic acid (FQA), 4-FQA, ferulic acid (FA) 4-sulfate, FA 4-glucuronide, feruloylglycine or 3-hydroxyhippuric acid (circled in blue).

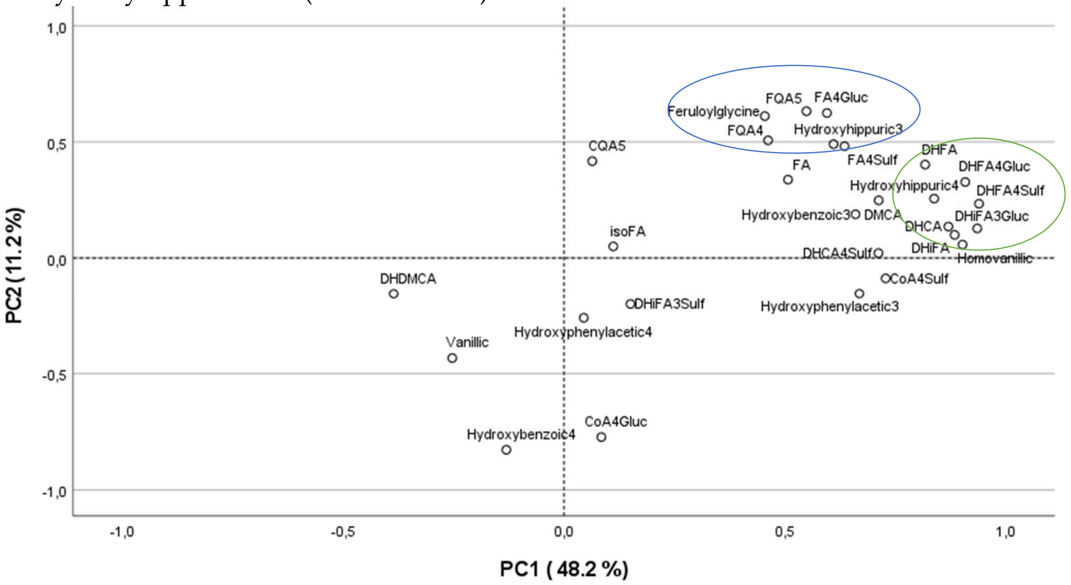

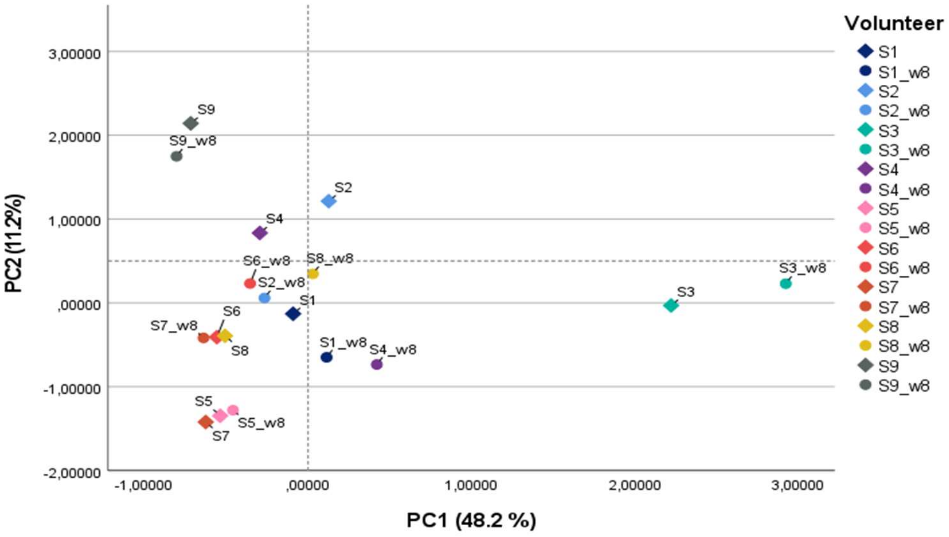

When individual scores with respect to each PC were determined for each participant at the beginning (week 0) and at the end of the intervention (week 8, *w8*), it was possible to observe how plasma accumulation profiles in week-8 did not differ much from baseline (points remaining

well grouped) except for **subject #3** (very positive values for PC1). This suggests high accumulation of PC1 linked compounds (e.g. DHFA, DHFA-4-glucuronide, DHFA-4-sulfate, DHFA-3-glucuronide, DHiFA) in this volunteer. Similarly, **subject #9** showed positive values for PC2 before and after supplementation (high presence of 5-FQA, 4-FQA, FA 4-sulfate, FA 4-glucuronide, feruloylglycine or 3-hydroxyhippuric acid), while **subject #5** exhibited low scores for PC2 (lower presence of these compounds).

<u>**URINE:**</u> Regarding urine samples, four PCs explained 61.8% of the total variability:

- PC1 (22.8% of total variability) was positively linked with an elevated excretion of caffeoylquinic acid (CQA) isoforms (3-CQA, 5-CQA), 5-FQA, *iso*ferulic acid (iFA), iFA 3-sulfate, caffeic acid (CA), CA 3-sulfate, DHCA 3-sulfate (circled in green), while it was inversely correlated with dihydrocoumarolyquinic acid (DHCoQA) and 5-dihydroferuloilquinic acid (DHFQA).
- PC2 (14.8% of total variability) had positive component loadings from feruloylglycine and DHFA 4- glucuronide (circled in blue).

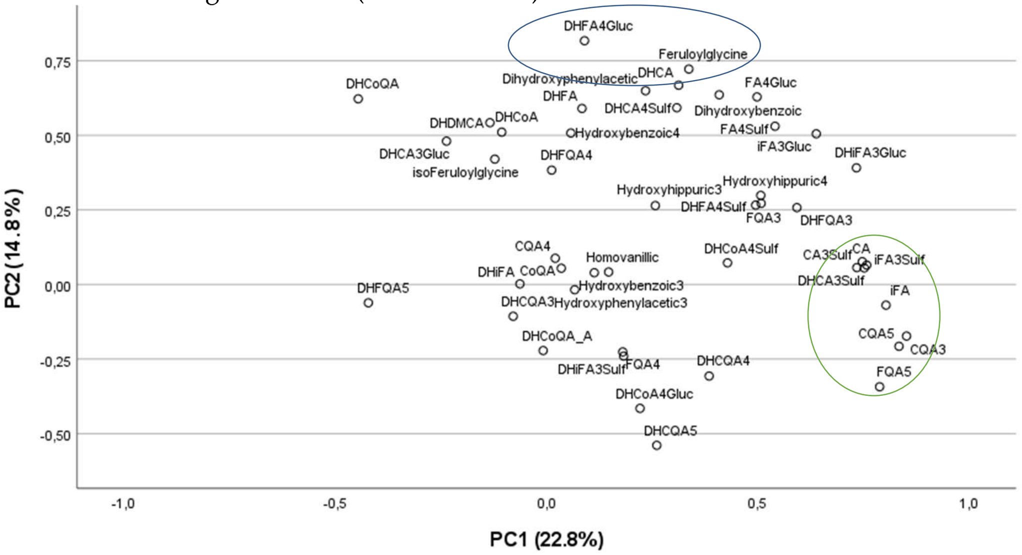

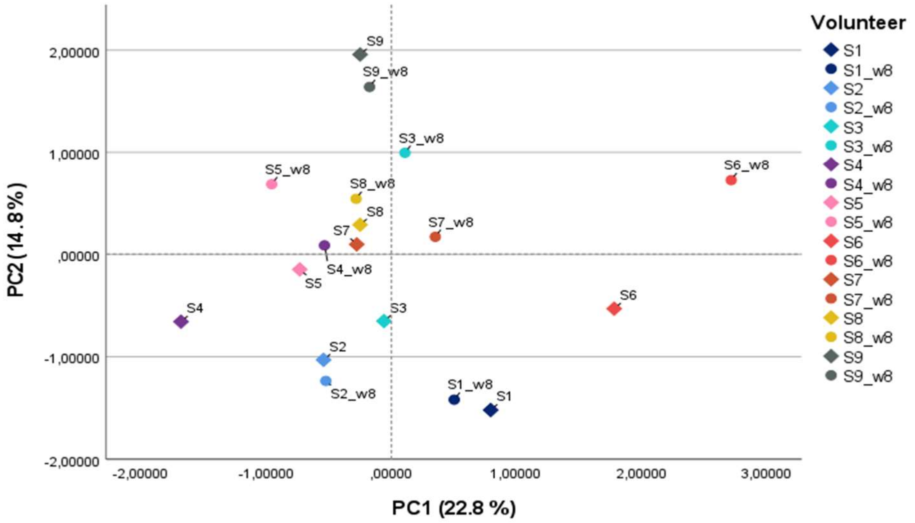

When the individual scores were determined, **subject #6** excretion profiles at week 0 and week 8 showed positive values for PC1 (high excretion of 3-CQA, 5-CQA, 5-FQA, iFA, iFA-3-sulfate or CA). Similarly, **subject #9** displayed high scores for PC2 (high excretion of dihydroferulic acid 4-glucuronide and feruloylglycine), while **subjects #1 and #2** had low scores for PC2 (limited excretion of DHFA 4-glucuronide and feruloylglycine).

<u>**FECES:**</u> Finally, in faecal samples, four PCs explained 64.6% of the total variability:

- PC1 (22.8% of total variability) was associated with an elevated excretion of 4-hydroxy-3-methoxybenzoic acid, 4′-hydroxy-3′-methoxyphenilacetic acid, 3-hydroxybenzoic acid, 3′-hydroxyphenylacetic acid and coumaric acid (CoA) (circled in green).
- PC2 (16.8% of total variability) was positive loaded to FA, DHFA, dihydrocoumaric acid (DHCoA) and dihydro*iso*coumaric acid (DHiCoA) (circled in blue).

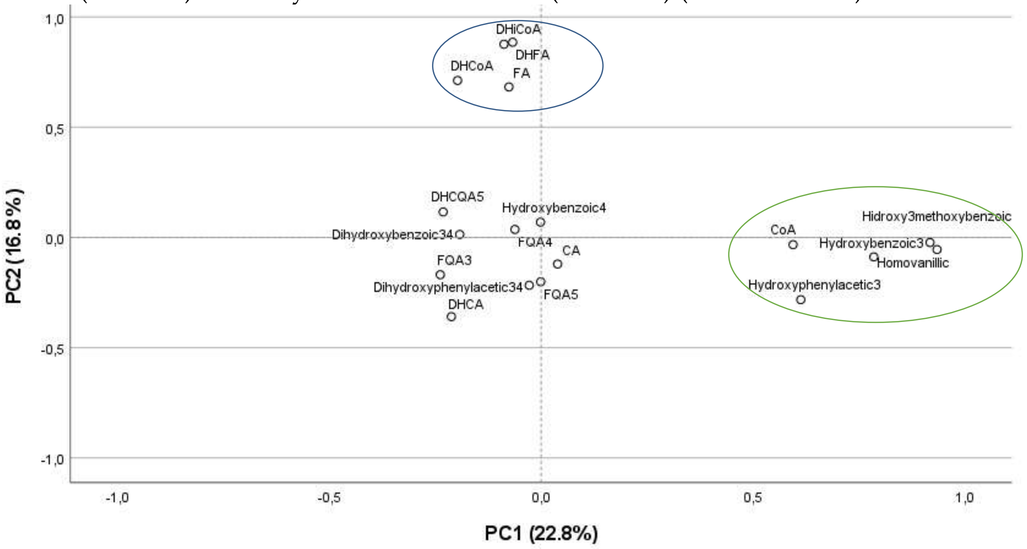

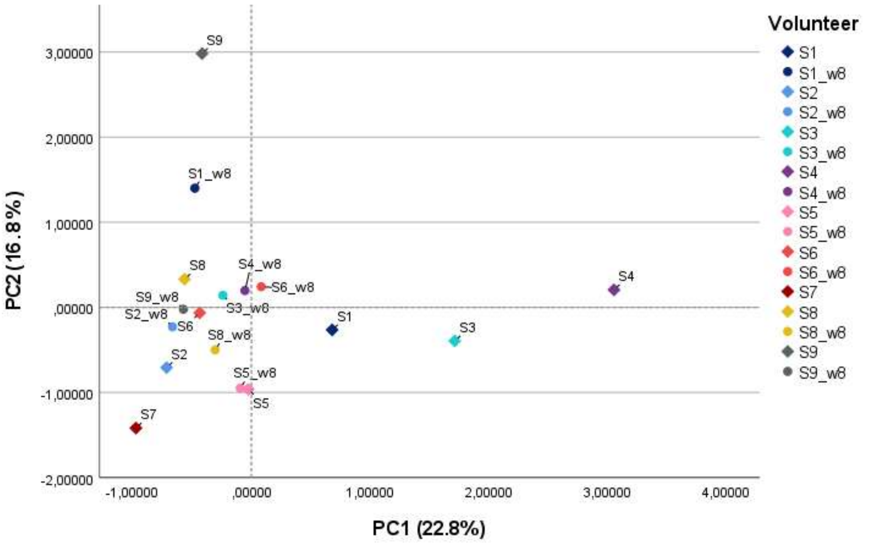

Individual scores indicated that faecal excretion profiles differed considerably from week 0 to week 8 in some volunteers. For instance, the excretions of **subjects #4 and #3** at week 0 displayed high scores for PC1, as well as **subject #9** at week 0 showed high scores for PC2. However, in all these subjects this excretion pattern is not reflected in week 8.

Overall, the groups of phenolic metabolites that contributed most to the observed interindividual variability were distinct for each biofluid. In plasma samples the group with the strongest influence on interindividual variability were the reduced forms of ferulic acid; in urine samples were the quinic acid conjugates of caffeic and ferulic acids; in fecal samples were derived forms of hydroxyphenylacetic and hydroxybenzoic acids. Therefore, there was no clear pattern for all samples. This was notably reflected in the differences observed in the **subjects #3, #9, #6, #4, #5, #1** or **#2,** highlighting that the complexity of interindividual variability is a key factor affecting phenolic bioavailability in human studies.

Table S1 Quantities of polyphenols in the green coffee phenolic extract (GCPE) identified by HPLC-DAD.

Polyphenols	mg/g d.m. (%)
Caffeoylquinic acids	302 ± 1 (65.9%)
Coumaroylquinic acids	1.23 ± 0.09 (0.3%)
Feruloylquinic acids	35.0 ± 0.5 (7.6%)
Dimethoxycinnamoylquinic acids	0.35 ± 0.07 (0.1%)
Dicaffeoylquinic acids	87 ± 1 (19.1%)
Caffeoylferuloylquinic acids	21.2 ± 0.4 (4.6%)
Caffeoylshikimic acids	0.5 ± 0.1 (0.1%)
Coumaroylcaffeoylquinic acids	6.24 ± 0.07 (1.4%)
Caffeoyl-glycosides	1.1 ± 0.1 (0.2%)
3′,4′-Dihydroxycinnamic acid (*Caffeic acid*)	0.60 ± 0.06 (0.1%)
Caffeoyl-*N*-tryptophan (mg/g)	2.57 ± 0.04 (0.6%)
**Total Hydroxycinnamic acids**	**458 ± 4**

Values are mean ± standard error (*n* =3). Data in parenthesis are the percentage of (poly)phenols with respect to the total amount quantified. d.m: dry matter

Table S2 Subject characteristics at baseline (week 0) and after 8 weeks of GCPE intervention.

	Week 0	Week 8	*p*-value
Glucose (mg/dL)	102 ± 7	106 ± 10	N.S.
Insulin (μUI/mL)	13 ± 2	13 ± 2	N.S.
HOMA-IR	3.4 ± 0.7	3.7 ± 0.8	N.S.
Total cholesterol (mg/dL)	210 ± 18	201 ± 11	N.S.
Triglycerides (mg/dL)	140 ± 42	120 ± 18	N.S.
HDL-Cholesterol (mg/dL)	49 ± 3	52 ± 2	N.S.
LDL-Cholesterol (mg/dL)	132 ± 13	126 ± 8	N.S.
BMI (kg/m^2)	31 ± 1	31 ± 1	N.S.
Weight (kg)	91 ± 5	90 ± 5	N.S.
Total Fat (%)	34 ± 2	34 ± 2	N.S.
Systolic Blood Pressure (mmHg)	131 ± 5	128 ± 5	N.S.
Diastolic Blood Pressure (mmHg)	87 ± 4	86 ± 3	N.S.

Values are mean ± standard error (*n* =9). HOMA-IR: homeostasis model assessment of insulin resistance; HDL: high density lipoprotein; LDL: low-density lipoprotein; BMI: body mass index. N.S.: non-significant differences between week 0 and week 8 (*p*-value>0.05)

Table S3 Chromatographic and spectrometric properties of the identified phenolic metabolites [1]

Identified Compound	RT (min)	Molecular formula	[M-H]⁻	Fragment MS²	Biofluid
**Caffeic acid metabolites**					
3-Caffeoylquinic acid	5.0	$C_{16}H_{18}O_9$	353.0878	191	U
5-Caffeoylquinic acid	7.6	$C_{16}H_{18}O_9$	353.0878	191	P, U
4-Caffeoylquinic acid	12.6	$C_{16}H_{18}O_9$	353.0878	191	U
3′,4′-Dihydroxycinnamic acid (*Caffeic acid, CA*)	8.1	$C_9H_8O_4$	179.0350	135	U, F
4′Hydroxycinnamic acid-3′-sulfate (*CA-3′-sulfate*)	8.1	$C_9H_8O_7S$	258.9918	179; 135	U
3-(3′,4′-Dihydroxyphenyl)propanoic acid (*Dihydrocaffeic acid, DHCA*)	6.8	$C_9H_{10}O_4$	181.0506	137	P, U, F
3-(4′-Hydroxyphenyl)propanoic acid-3′-glucuronide (*DHCA-3′-glucuronide*)	4.1	$C_{15}H_{18}O_{10}$	357.0827	181; 137	U
3-(3′-Hydroxyphenyl)propanoic acid-4′-sulfate (*DHCA-4′-sulfate*)	5.8	$C_9H_{10}O_7S$	261.0074	181	P, U
3-(4′-Hydroxyphenyl)propanoic acid-3′-sulfate (*DHCA-3′-sulfate*)	7.5	$C_9H_{10}O_7S$	261.0074	181	U
3-Dihydrocaffeoylquinic acid	9.5	$C_{16}H_{20}O_9$	355.1035	181	U
5-Dihydrocaffeoylquinic acid	14.6	$C_{16}H_{20}O_9$	355.1035	181	U, F
4-Dihydrocaffeoylquinic acid	15.5	$C_{16}H_{20}O_9$	355.1035	181	U
**Ferulic acid metabolites**					
3-Feruloylquinic acid	8.4	$C_{17}H_{20}O_9$	367.1035	191	U, F
5-Feruloylquinic acid	10.8	$C_{17}H_{20}O_9$	367.1035	191	P, U, F
4-Feruloylquinic acid	11.4	$C_{17}H_{20}O_9$	367.1035	191	P, U, F
4′-Hydroxy-3′-methoxycinnamic acid (*Ferulic acid, FA*)	13.1	$C_{10}H_{10}O_4$	193.0506	134	P, F
3′-Hydroxy-4′-methoxycinnamic acid (*isoFerulic acid, iFA*)	13.8	$C_{10}H_{10}O_4$	193.0506	134	P, U
3′-Methoxycinnamic acid-4′-glucuronide (*FA-4′-glucuronide*)	6.1	$C_{16}H_{18}O_{10}$	369.0827	193; 134	P,U
4′-Methoxycinnamic acid-3′-glucuronide (*iFA-3′-glucuronide*)	9.4	$C_{16}H_{18}O_{10}$	369.0827	193; 134	U
3′-Methoxycinnamic acid-4′-sulfate (*FA-4′-sulfate*)	8.3	$C_{10}H_{10}O_7S$	273.0074	193; 134	P, U
4′-Methoxycinnamic acid-3′-sulfate (*iFA-3′-sulfate*)	9.6	$C_{10}H_{10}O_7S$	273.0074	193; 134	U
3-(4′-Hydroxy-3′-methoxyphenyl)propanoic acid (*Dihydroferulic acid, DHFA*)	9.3	$C_{10}H_{12}O_4$	195.0663	136	P, U, F
3-(3′-Hydroxy-4′-methoxyphenyl)propanoic acid (*Dihydroisoferulic acid, DHiFA*)	12.2	$C_{10}H_{12}O_4$	195.0663	136	P, U
3-(3′-Methoxyphenyl)propanoic acid-4′-glucuronide (*DHFA- 4′-glucuronide*)	8.1	$C_{16}H_{20}O_{10}$	371.0984	195; 136	P, U
3-(4′-Methoxyphenyl)propanoic acid-3′-glucuronide (*DHiFA- 3′-glucuronide*)	9.6	$C_{16}H_{20}O_{10}$	371.0984	195; 136	P, U
3-(3′-Methoxyphenyl)propanoic acid-4′-sulfate (*DHFA- 4′-sulfate*)	8.2	$C_{10}H_{12}O_7S$	275.0231	195; 136	P, U
3-(4′-Methoxyphenyl)propanoic acid-3′-sulfate (*DHiFA- 3′-sulfate*)	12.4	$C_{10}H_{12}O_7S$	275.0231	195; 136	P, U
3-Dihydroferuloylquinic acid	13.1	$C_{17}H_{22}O_9$	369.1191	195	U
5-Dihydroferuloylquinic acid	13.4	$C_{17}H_{22}O_9$	369.1191	195	U
4-Dihydroferuloylquinic acid	14.3	$C_{17}H_{22}O_9$	369.1191	195	U
Feruloylglycine	10.0	$C_{12}H_{13}O_5N$	250.0721	191; 134	P, U
*iso*Feruloylglycine	11.7	$C_{12}H_{13}O_5N$	250.0721	191; 134	U
**Coumaric acid metabolites**					
4′-Hydroxycinnamic acid *(Coumaric acid, CoA)*	11.7	$C_9H_8O_3$	163.0401	163	F
Coumaroylquinic acid	17.5	$C_{16}H_{18}O_8$	337.0929	163	U
Cinnamic acid-4′-glucuronide (*Coumaric acid-4′-glucuronide, CoA-4′-glucuronide*)	12.3	$C_{15}H_{16}O_9$	339.0722	163	P
Cinnamic acid-4′-sulfate (*CoA-4′-sulfate*)	18.5	$C_9H_8O_6S$	242.9969	163	P
3-(4′-Hydroxyphenyl)propanoic acid (*Dihydrocoumaric acid, DHCoA*)	12.6	$C_9H_{10}O_3$	165.0557	121	U, F
3-(3′-Hydroxyphenyl)propanoic acid *(Dihydroisocoumaric acid, DHiCoA)*	11.2	$C_9H_{10}O_3$	165.0557	121	F
3-(Phenyl)propanoic acid-4′-glucuronide (*DHCoA-4′-glucuronide*)	8.8	$C_{15}H_{18}O_9$	341.0878	165	U
3-(Phenyl)propanoic acid-4′-sulfate (*DHCoA-4′-sulfate*)	8.8	$C_9H_{10}O_6S$	245.0125	165	U
Dihydrocoumaroylquinic acid	16.2	$C_{16}H_{20}O_8$	339.1085	165	U
Dihydrocoumaroylquinic acid	18.0	$C_{16}H_{20}O_8$	339.1085	165	U
**Dimethoxycinnamic acid metabolites**					
3′,4′-Dimethoxycinnamic acid	15.1	$C_{11}H_{12}O_4$	207.0652	103	P
3-(3′,4′-Dimethoxyphenyl)propanoic acid (*Dihydrodimethoxycinnamic acid*)	15.1	$C_{11}H_{14}O_4$	209.0819		P, U
**Phenolic acids**					
3′,4′-Dihydroxyphenylacetic acid	6.1	$C_8H_8O_4$	167.0350	123	U, F
4′-Hydroxy-3′-methoxyphenylacetic acid	9.8	$C_9H_{10}O_4$	181,0506	137	P, U
4′-Hydroxyphenylacetic acid	10.1	$C_8H_8O_3$	151,0401	107	P
3′-Hydroxyphenylacetic acid	8.0	$C_8H_8O_3$	151,0401	107	P, U, F
3,4-Dihydroxybenzoic acid	5.3	$C_7H_6O_4$	153,0193	109	U, F
4-Hydroxy-3-methoxybenzoic acid	8.9	$C_8H_8O_4$	167.0350	123	P, F
4-Hydroxybenzoic acid	10.0	$C_7H_6O_3$	137.0244	93	P, U, F
3-Hydroxybenzoic acid	8.0	$C_7H_6O_3$	137.0244	93	P, U, F
4′-Hydroxyhippuric acid	14.2	$C_9H_9O_4N$	194.0459	100	P, U
3′-Hydroxyhippuric acid	11.0	$C_9H_9O_4N$	194.0459	100	P, U

[1] RT: retention time.

Table S4 Urine kinetic profile of phenolic metabolites after the ingestion of the GCPE nutraceutical at the beginning (week 0) and at the end of the intervention (week 8).

	Week 0			Week 8		
Metabolite	C_{max} (μM)	T_{max} range (h)	AUC_{0-24h} (μM min^{-1})	C_{max} (μM)	T_{max} range (h)	AUC_{0-24h} (μM min^{-1})
**Intestinal Absorption**						
3-Caffeoylquinic acid	0.17 ± 0.04	(0 – 3)	0.9 ± 0.3	0.15 ± 0.02	(0 – 6)	1.0 ± 0.2
5-Caffeoylquinic acid	0.27 ± 0.05	(0 – 3)	1.4 ± 0.3	0.24 ± 0.03	(0 – 6)	1.5 ± 0.3
4-Caffeoylquinic acid	0.2 ± 0.1	(0 – 24)	1 ± 1	0.17 ± 0.07	(0 – 11)	1.4 ± 0.8
3-Feruloylquinic acid	0.25 ± 0.06	(0 – 6)	1.4 ± 0.4	0.27 ± 0.07	(0 – 6)	1.8 ± 0.5
5-Feruloylquinic acid	1.2 ± 0.3	(0 – 6)	7 ± 1	1.1 ± 0.2	(0 – 6)	8 ± 1
4-Feruloylquinic acid	0.3 ± 0.1	(0 – 6)	1.6 ± 0.6	0.27 ± 0.08	(0 – 6)	1.8 ± 0.7
Coumaroylquinic acid	0.07 ± 0.02	(0 – 24)	1.2 ± 0.4	0.08 ± 0.02	(0 – 24)	0.8 ± 0.2
3′,4′-Dihydroxycinnamic acid (*Caffeic acid, CA*)	0.24 ± 0.06	(0 – 9)	2.5 ± 0.4	0.34 ± 0.08	(0 – 11)	3.7 ± 0.9
4′Hydroxycinnamic acid-3′-sulfate (*CA-3′-sulfate*)	2.2 ± 0.5	(0 – 6)	22 ± 4	3.1 ± 0.8	(0 – 11)	35 ± 9
3′-Hydroxy-4′-methoxycinnamic acid (*isoFerulic acid, iFA*)	1.6 ± 0.4	(0 – 6)	8 ± 2	1.7 ± 0.2	(0 – 6)	13 ± 2
3′-Methoxycinnamic acid-4′-glucuronide (*FA-4′-glucuronide*)	1.5 ± 0.2	(0 – 24)	45 ± 23	2.3 ± 0.5	(0 – 24)	30 ± 6
4′-Methoxycinnamic acid-3′-glucuronide (*iFA-3′-glucuronide*)	2.1 ± 0.3	(0 – 11)	29 ± 3*	4 ± 1	(0 – 11)	51 ± 12*
3′-Methoxycinnamic acid-4′-sulfate (*FA-4′-sulfate*)	15 ± 2	(0 – 24)	225 ± 33	21 ± 5	(0 – 24)	283 ± 55
4′-Methoxycinnamic acid-3′-sulfate (*iFA-3′-sulfate*)	0.8 ± 0.2	(0 – 11)	8 ± 2*	1.4 ± 0.5	(0 – 11)	19 ± 7*
**Colonic absorption**						
3-(3′,4′-Dihydroxyphenyl)propanoic acid (*Dihydrocaffeic acid, DHCA*)	7.7 ± 0.7**	(11 – 24)	96 ± 16*	10 ± 1**	(11 – 24)	125 ± 18*
3-(4′-Hydroxy-3′-methoxyphenyl)propanoic acid (*Dihydroferulic, DHFA*)	0.19 ± 0.09	(3 – 9)	2 ± 1	0.4 ± 0.2	(3 – 9)	4 ± 2
3-(3′-Hydroxy-4′-methoxyphenyl)propanoic acid (*Dihydroisoferulic, DHiFA*)	2.7 ± 0.6	(6 – 24)	27 ± 4	2.5 ± 0.7	(9 – 24)	26 ± 6
3-(4′-Hydroxyphenyl)propanoic acid (*Dihydrocoumaric acid, DHCoA*)	1.7 ± 0.5	(11 – 24)	21 ± 7	2.5 ± 0.6	(9 – 24)	27 ± 8
3-(3′,4′-Dimethoxyphenyl)propanoic acid (*Dihydrodimethoxycinnamic acid*)	0.3 ± 0.1	(11– 24)	4.1 ± 0.6	0.32 ± 0.04	(11 – 24)	4.3 ± 0.5
3-(4′-Hydroxyphenyl)propanoic acid-3′-glucuronide (*DHCA-3′-glucuronide*)	0.23 ± 0.04	(11 – 24)	3.3 ± 0.7	0.25 ± 0.04	(9 – 11)	3.9 ± 0.9
3-(3′-Hydroxyphenyl)propanoic acid-4′-sulfate (*DHCA-4′-sulfate*)	2.2 ± 0.8	(11– 24)	31 ± 12	2.8 ± 0.7	(11 – 24)	35 ± 9
3-(4′-Hydroxyphenyl)propanoic acid-3′-sulfate (*DHCA-3′-sulfate*)	7 ± 2	(11 – 24)	68 ± 19	11 ± 4	(9 – 24)	109 ± 42
3-(3′-Methoxyphenyl)propanoic acid-4′-glucuronide (*DHFA- 4′-glucuronide*)	2.2 ± 0.4	(9 – 24)	25 ± 3	3.0 ± 0.7	(9 – 24)	32 ± 7
3-(4′-Methoxyphenyl)propanoic acid-3′-glucuronide (*DHiFA- 3′-glucuronide*)	2.5 ± 0.6	(9 – 11)	23 ± 4	4 ± 1	(9 – 11)	33 ± 10
3-(3′-Methoxyphenyl)propanoic acid-4′-sulfate (*DHFA- 4′-sulfate*)	8 ± 3	(9 – 24)	80 ± 19	11 ± 4	(9 – 24)	116 ± 38
3-(4′-Methoxyphenyl)propanoic acid-3′-sulfate (*DHiFA- 3′-sulfate*)	1.7 ± 0.7	(11 – 24)	20 ± 7	2.2 ± 0.8	(11 – 24)	19 ± 5
3-(Phenyl)propanoic acid-4′-glucuronide (*DHCoA-4′-glucuronide*)	1.2 ± 0.2	(11 – 24)*	15 ± 2	1.8 ± 0.7	(9 – 24)*	23 ± 4
3-(Phenyl)propanoic acid-4′-sulfate (*DHCoA-4′-sulfate*)	12 ± 3	(11 – 24)*	161 ± 56*	21 ± 7	(9 – 24)*	251 ± 65*
3-Dihydrocaffeoylquinic acid	0.17 ± 0.04	(11 – 24)	2.7 ± 0.8	0.21 ± 0.07	(11 – 24)	2.3 ± 0.6
5-Dihydrocaffeoylquinic acid	0.03 ± 0.02	(3 – 9)	0.3 ± 0.1	0.018 ± 0.009	(3 – 11)	0.2 ± 0.1
4-Dihydrocaffeoylquinic acid	0.030 ± 0.007	(11- 24)	0.5 ± 0.1	0.03 ± 0.01	(9 – 24)	0.4 ± 0.1
3-Dihydroferuloylquinic acid	0.17 ± 0.08	(9 – 24)	1.6 ± 0.6**	0.29 ± 0.07	(6 – 11)	4 ± 1**

5-Dihydroferuloylquinic acid	0.19 ± 0.1	(3 – 11)	1.9 ± 0.8	0.13 ± 0.05	(3 – 11)	1.8 ± 0.8
4-Dihydroferuloylquinic acid	0.03 ± 0.01	(3 – 11)	0.4 ± 0.1	0.04 ± 0.02	(3 – 9)	0.4 ± 0.2
Dihydrocoumaroylquinic acid	0.4 ± 0.1	(11 – 24)	3.3 ± 0.7	0.5 ± 0.1	(9 – 24)	4 ± 1
Dihydrocoumaroylquinic acid	0.13 ± 0.05	(11 – 24)	2 ± 1	0.15 ± 0.07	(11 – 24)	4 ± 1
Feruloylglycine	8 ± 2	(9 – 11)	173 ± 101	10 ± 3	(6 – 11)	108 ± 33
*Iso*Feruloylglicine	0.22 ± 0.01	(9 – 24)	3.2 ± 0.2	0.23 ± 0.03	(6 – 24)	3.1 ± 0.6
**Other metabolites**						
3′,4′-Dihydroxyphenylacetic acid	0.36 ± 0.05	(11 – 24)	4.7 ± 0.8	0.41 ± 0.07	(9 – 24)	5.2 ± 0.7
4′-Hydroxy-3′-methoxyphenylacetic acid	3.1 ± 0.4	(11 – 24)	48 ± 5	3.7 ± 0.9	(9 – 24)	49 ± 8
3′-Hydroxyphenylacetic acid	3 ± 1	(9 – 24)	45 ± 12	2.0 ± 0.4	(9 – 24)	33 ± 8
3,4-Dihydroxybenzoic acid	0.050 ± 0.006	(11 – 24)	0.6 ± 0.1	0.07 ± 0.02	(6 – 11)	0.8 ± 0.2
4-Hydroxybenzoic acid	0.4 ± 0.1	(9 – 24)	4.9 ± 0.4	0.25 ± 0.02	(11 – 24)	4.3 ± 0.4
3-Hydroxybenzoic acid	0.23 ± 0.02	(11 – 24)	3.7 ± 0.4	0.27 ± 0.05	(9 – 24)	4.0 ± 0.7
4′-Hydroxyhippuric acid	3.3 ± 0.6	(11 – 24)	49 ± 8	3.5 ± 0.9	(9 – 24)	55 ± 12
3′-Hydroxyhippuric acid	13 ± 2	(9 – 24)	169 ± 27	16 ± 3	(9 – 24)	204 ± 36

Values represent mean ± standard error (n = 9). *$p < 0.05$ **$p < 0.01$ week 0 vs. week 8.

Table S5 Amount of urine metabolites excreted 0-24 h after consumption of the GCPE nutraceutical at the beginning of the intervention (week 0)

Metabolites	0 h	0- 3 h	3-6 h	6-9 h	9-11 h	11-24 h	TOTAL (moles)
**Intestinal absorption**							
3-Caffeoylquinic acid	N.D.	0.11 ± 0.02	0.08 ± 0.03	0.022 ± 0.009	0.003 ± 0.002	N.D.	**0.21 ± 0.05**
5-Caffeoylquinic acid	N.D.	0.18 ± 0.04	0.12 ± 0.04	0.03 ± 0.01	0.004 ± 0.002	N.D.	**0.33 ± 0.06**
4-Caffeoylquinic acid	N.D.	N.D.	N.D.	0.03 ± 0.02	0.05 ± 0.04	0.05 ± 0.02	**0.13 ± 0.07**
3-Feruloylquinic acid	N.D.	0.15 ± 0.04	0.10 ± 0.04	0.021 ± 0.006	0.007 ± 0.004	0.010 ± 0.005	**0.29 ± 0.07**
5-Feruloylquinic acid	N.D.	0.8 ± 0.2	0.5 ± 0.1	0.13 ± 0.04	0.03 ± 0.01	0.09 ± 0.01	**1.5 ± 0.2**
4-Feruloylquinic acid	N.D.	0.2 ± 0.1	0.15 ± 0.05	0.02 ± 0.01	0.003 ± 0.002	N.D.	**0.4 ± 0.2**
Coumaroylquinic acid	N.D.	0.03 ± 0.01	0.015 ± 0.003	0.017 ± 0.006	0.015 ± 0.004	0.06 ± 0.02	**0.14 ± 0.03**
3′,4′-Dihydroxycinnamic acid (*Caffeic acid, CA*)	N.D.	0.13 ± 0.03	0.08 ± 0.02	0.08 ± 0.03	0.023 ± 0.008	0.12 ± 0.02	**0.42 ± 0.06**
4′Hydroxycinnamic acid-3′-sulfate (*CA-3′-sulfate*)	N.D.	1.2 ± 0.3	0.7 ± 0.2	0.8 ± 0.2	0.21 ± 0.06	0.9 ± 0.2	**3.7 ± 0.5**
3′-Hydroxy-4′-methoxycinnamic acid (*isoFerulic acid, iFA*)	N.D.	1.0 ± 0.2	0.35 ± 0.07	0.27 ± 0.07	0.04 ± 0.03	0.04 ± 0.02	**1.7 ± 0.2**
3′-Methoxycinnamic acid-4′-glucuronide (*FA-4′-glucuronide*)	N.D.	0.5 ± 0.1	0.42 ± 0.07	0.7 ± 0.2	0.44 ± 0.09	1.3 ± 0.3	**3.4 ± 0.4**
4′-Methoxycinnamic acid-3′-glucuronide (*iFA-3′-glucuronide*)	N.D.	0.9 ± 0.2	0.8 ± 0.2	1.1 ± 0.3	0.49 ± 0.09	1.1 ± 0.2	**4.4 ± 0.5**
3′-Methoxycinnamic acid-4′-sulfate (*FA-4′-sulfate*)	N.D.	5 ± 2	3.2 ± 0.9	7 ± 2	3.6 ± 0.7	13 ± 2	**32 ± 5**
4′-Methoxycinnamic acid-3′-sulfate (*iFA-3′-sulfate*)	N.D.	0.4 ± 0.1	0.07 ± 0.04	0.24 ± 0.08	0.09 ± 0.03	0.4 ± 0.1	**1.1 ± 0.3**
**TOTAL – Intestinal absorption**	**N.D.**	**12 ± 3**	**7 ± 1**	**10 ± 2**	**5.0 ± 0.9**	**17 ± 3**	**50 ± 6**
**Colonic absorption**							
3-(3′,4′-Dihydroxyphenyl)propanoic acid (*Dihydrocaffeic acid, DHCA*)	3 ± 1	0.5 ± 0.1	0.37 ± 0.09	2.3 ± 0.8	2.7 ± 0.6	7 ± 1	**16 ± 2**
3-(4′-Hydroxy-3′-methoxyphenyl) propanoic acid (*Dihydroferulic, DHFA*)	0.2 ± 0.1	0.03 ± 0.02	0.007 ± 0.007	0.08 ± 0.04	0.05 ± 0.02	0.11 ± 0.05	**0.5 ± 0.2**
3-(3′-Hydroxy-4′-methoxyphenyl) propanoic acid (*Dihydroisoferulic, DHiFA*)	N.D.	0.26 ± 0.04	0.25 ± 0.08	1.2 ± 0.3	0.8 ± 0.2	1.2 ± 0.09	**3.7 ± 0.4**
3-(4′-Hydroxyphenyl)propanoic acid (*Dihydrocoumaric acid, DHCoA*)	1.1 ± 0.4	0.16 ± 0.04	0.09 ± 0.02	0.5 ± 0.2	0.6 ± 0.2	1.4 ± 0.4	**4 ± 1**
3-(3′,4′-Dimethoxyphenyl)propanoic acid (*Dihydrodimethoxycinnamic acid*)	N.D.	0.08 ± 0.02	0.08 ± 0.01	0.2 ± 0.1	0.09 ± 0.02	0.27 ± 0.06	**0.7 ± 0.2**
3-(4′-Hydroxyphenyl)propanoic acid-3′-glucuronide (*DHCA-3′-glucuronide*)	N.D.	0.09 ± 0.03	0.07 ± 0.02	0.08 ± 0.04	0.04 ± 0.02	0.30 ± 0.09	**0.6 ± 0.2**
3-(3′-Hydroxyphenyl)propanoic acid-4′-sulfate (*DHCA-4′-sulfate*)	3.5 ± 0.8	0.4 ± 0.1	0.20 ± 0.04	0.5 ± 0.2	0.5 ± 0.2	2.6 ± 0.8	**8 ± 2**
3-(4′-Hydroxyphenyl)propanoic acid-3′-sulfate (*DHCA-3′-sulfate*)	N.D.	0.32 ± 0.07	0.26 ± 0.07	2.5 ± 0.9	2.0 ± 0.7	4 ± 1	**9 ± 2**
3-(3′-Methoxyphenyl)propanoic acid-4′-glucuronide (*DHFA- 4′-glucuronide*)	1.4 ± 0.8	0.15 ± 0.05	0.16 ± 0.05	1.0 ± 0.4	0.8 ± 0.1	1.2 ± 0.2	**5 ± 1**
3-(4′-Methoxyphenyl)propanoic acid-3′-glucuronide (*DHiFA- 3′-glucuronide*)	N.D.	0.12 ±0.04	0.16 ± 0.06	1.1 ± 0.4	0.7 ± 0.2	0.8 ± 0.2	**2.9 ± 0.5**
3-(3′-Methoxyphenyl)propanoic acid-4′-sulfate (*DHFA- 4′-sulfate*)	N.D.	0.5 ± 0.1	0.5 ± 0.1	3.4 ± 1.2	2.1 ± 0.5	3.5 ± 0.8	**10 ± 2**
3-(4′-Methoxyphenyl)propanoic acid-3′-sulfate (*DHiFA- 3′-sulfate*)	N.D.	0.18 ± 0.06	0.10 ± 0.04	0.4 ± 0.1	0.2 ± 0.1	1.9 ± 0.8	**3 ± 1**
3-(Phenyl)propanoic acid-4′-glucuronide (*DHCoA-4′-glucuronide*)	N.D.	0.17 ± 0.06	0.10 ± 0.03	0.26 ± 0.09	0.28 ± 0.07	1.3 ± 0.2	**2.1 ± 0.3**
3-(Phenyl)propanoic acid-4′-sulfate (*DHCoA-4′-sulfate*)	N.D.	0.9 ± 0.4	0.7 ± 0.1	3 ± 1	3 ± 1	12 ± 4	**21 ± 6**
3-Dihydrocaffeoylquinic acid	N.D.	0.06 ± 0.02	0.033 ± 0.005	0.05 ± 0.02	0.04 ± 0.01	0.17 ± 0.04	**0.35 ± 0.07**
5-Dihydrocaffeoylquinic acid	N.D.	0.012 ± 0.006	0.005 ± 0.003	0.003 ± 0.002	0.003 ± 0.002	0.010 ± 0.004	**0.03 ± 0.02**
4-Dihydrocaffeoylquinic acid	N.D.	0.011 ± 0.004	0.005 ± 0.002	0.005 ± 0.002	0.006 ± 0.002	0.036 ± 0.008	**0.06 ± 0.02**
3-Dihydroferuloylquinic acid	N.D.	0.05 ± 0.01	0.034 ± 0.006	0.029 ± 0.006	0.019 ± 0.005	0.16 ± 0.09	**0.3 ± 0.1**
5-Dihydroferuloylquinic acid	N.D.	0.12 ± 0.08	0.023 ± 0.004	0.027 ± 0.009	0.013 ± 0.004	0.10 ± 0.04	**0.3 ± 0.1**
4-Dihydroferuloylquinic acid	N.D.	0.011 ± 0.004	0.008 ± 0.003	0.013 ± 0.005	0.005 ± 0.003	0.019 ± 0.009	**0.06 ± 0.02**
Dihydrocoumaroylquinic acid	N.D.	0.005 ± 0.002	0.005 ± 0.003	0.2 ± 0.1	0.12 ± 0.05	0.14 ± 0.04	**0.5 ± 0.1**
Dihydrocoumaroylquinic acid	N.D.	0.04 ± 0.02	0.017 ± 0.004	0.018 ± 0.005	0.022 ± 0.009	0.12 ± 0.05	**0.22 ± 0.08**

Feruloylglycine	7 ± 3	1.0 ± 0.2	0.9 ± 0.3	4 ± 1	2.6 ± 0.9	3.9 ± 0.9	**19 ± 5**
*Iso*Feruloylglicine	N.D.	0.04 ± 0.01	0.035 ± 0.009	0.08 ± 0.02	0.11 ± 0.01	0.16 ± 0.03	**0.44 ± 0.04**
**TOTAL – Colonic absorption**	**16 ± 6**	**5 ± 1**	**4.0 ± 0.7**	**21 ± 6**	**17 ± 4**	**42 ± 8**	**106 ± 16**
**Other microbial metabolites**							
3´,4´-Dihydroxyphenylacetic acid	0.42 ± 0.08	0.12 ± 0.03	0.08 ± 0.03	0.11 ± 0.05	0.06 ± 0.02	0.38 ± 0.04	**1.2 ± 0.2**
4´-Hydroxy-3´-methoxyphenylacetic acid	5.9 ± 0.6	1.3 ± 0.2	0.9 ± 0.2	0.9 ± 0.2	0.7 ± 0.1	3.6 ± 0.3	**13.2 ± 0.9**
3´-Hydroxyphenylacetic acid	6 ± 1	1.6 ± 0.5	1.0 ± 0.2	0.8 ± 0.2	0.53 ± 0.09	2.5 ± 0.7	**12 ± 3**
3,4-Dihydroxybenzoic acid	0.04 ± 0.01	0.016 ± 0.005	0.007 ± 0.003	0.017 ± 0.005	0.018 ± 0.006	0.035 ± 0.009	**0.13 ± 0.02**
4-Hydroxybenzoic acid	0.39 ± 0.05	0.3 ± 0.2	0.11 ± 0.02	0.10 ± 0.02	0.09 ± 0.01	0.29 ± 0.04	**1.3 ± 0.2**
3-Hydroxybenzoic acid	0.37 ± 0.04	0.09 ± 0.02	0.05 ± 0.01	0.08 ± 0.02	0.064 ± 0.009	0.26 ± 0.02	**0.91 ± 0.07**
4´-Hydroxyhippuric acid	7 ± 1	1.3 ± 0.3	0.7 ± 0.1	1.0 ± 0.3	0.8 ± 0.2	3.8 ± 0.9	**14 ± 2**
3´-Hydroxyhippuric acid	8 ± 3	3 ± 2	1.1 ± 0.2	4 ± 1	5 ± 1	11 ± 2	**32 ± 5**
**TOTAL – Other microbial metabolites**	**27 ± 4**	**8 ± 2**	**3.8 ± 0.6**	**7 ± 2**	**7 ± 1**	**22 ± 2**	**75 ± 7**
**TOTAL Colonic + other microbial met.**	**43 ± 10**	**13 ± 3**	**8 ± 1**	**28 ± 8**	**24 ± 5**	**64 ± 10**	**181 ± 21**
**TOTAL INTESTINAL+COLONIC+OTHERS**	**43 ± 10**	**24 ± 6**	**14 ± 2**	**39 ± 10**	**29 ± 6**	**81 ± 11**	**231 ± 26**

Values represent mean ± standard error (n = 9). N.D.: Not detected.

Table S6 Amount of urine metabolites excreted 0-24 h after consumption of the GCPE nutraceutical at the end of the intervention (week 8).

Metabolites	0 h	0- 3 h	3-6 h	6-9 h	9-11 h	11-24 h	TOTAL (moles)
**Intestinal absorption**							
3-Caffeoylquinic acid	N.D.	0.10 ± 0.02	0.045 ± 0.008	0.028 ± 0.007	0.008 ± 0.005	N.D.	**0.18 ± 0.03**
5-Caffeoylquinic acid	N.D.	0.18 ± 0.04	0.08 ± 0.01	0.033 ± 0.008	0.009 ± 0.007	0.006 ± 0.006	**0.33 ± 0.04**
4-Caffeoylquinic acid	N.D.	0.01 ± 0.01*	0.008±0.005*	0.06 ± 0.03	0.05 ± 0.03	0.02 ± 0.01	**0.15 ± 0.07**
3-Feruloylquinic acid	N.D.	0.19 ± 0.04	0.08 ± 0.02	0.05 ± 0.01	0.014 ± 0.008	0.015 ± 0.007	**0.34 ± 0.07**
5-Feruloylquinic acid	N.D.	0.8 ± 0.1	0.39 ± 0.07	0.20 ± 0.04	0.05 ± 0.03	0.08 ± 0.02	**1.5 ± 0.2**
4-Feruloylquinic acid	N.D.	0.21 ± 0.08	0.13 ± 0.03	0.03 ± 0.01	N.D.*	0.02 ± 0.02	**0.4 ± 0.1**
Coumaroylquinic acid	N.D.	0.009 ± 0.003	0.007±0.001**	0.014 ± 0.002	0.012 ± 0.004	0.06 ± 0.02*	**0.10 ± 0.02***
3′,4′-Dihydroxycinnamic acid (*Caffeic acid, CA*)	N.D.	0.12 ± 0.03	0.06 ± 0.01	0.11 ± 0.02	0.06 ± 0.03	0.11 ± 0.03	**0.45 ± 0.09**
4′Hydroxycinnamic acid-3′-sulfate (*CA-3′-sulfate*)	N.D.	1.2 ± 0.2	0.6 ± 0.1	0.9 ± 0.3	0.7 ± 0.3	1.0 ± 0.3	**4.4 ± 0.8**
3′-Hydroxy-4′-methoxycinnamic acid (*isoFerulic acid, iFA*)	N.D.	1.2 ± 0.2	0.32 ± 0.04	0.39 ± 0.07	0.13 ± 0.07	0.09 ± 0.04	**2.1 ± 0.2**
3′-Methoxycinnamic acid-4′-glucuronide (*FA-4′-glucuronide*)	N.D.	0.6 ± 0.1	0.37 ± 0.05	0.8 ± 0.1	0.8 ± 0.3	1.3 ± 0.2	**3.7 ± 0.5**
4′-Methoxycinnamic acid-3′-glucuronide (*iFA-3′-glucuronide*)	N.D.	1.4 ± 0.3	0.9 ± 0.2	1.7 ± 0.3	1.0 ± 0.4	1.1 ± 0.3	**6 ± 1**
3′-Methoxycinnamic acid-4′-sulfate (*FA-4′-sulfate*)	N.D.	4 ± 2	3.4 ± 0.7	7 ± 1	7 ± 2	11 ± 2	**33 ± 5**
4′-Methoxycinnamic acid-3′-sulfate (*iFA-3′-sulfate*)	N.D.	0.34 ± 0.07	0.10 ± 0.03	0.32 ± 0.06	0.3 ± 0.1	1.0 ± 0.7	**2.1 ± 0.9**
**TOTAL – Intestinal absorption**	**N.D.**	**11 ± 2**	**7 ± 1**	**12 ± 2**	**11 ± 3**	**16 ± 4**	**59 ± 8**
**Colonic absorption**							
3-(3′,4′-Dihydroxyphenyl)propanoic acid (*Dihydrocaffeic acid, DHCA*)	15 ± 4**	1.0 ± 0.4*	1.4 ± 0.4**	3.8 ± 0.9	3.1 ± 0.8	6 ± 2	**31 ± 5****
3-(4′-Hydroxy-3′-methoxyphenyl) propanoic acid (*Dihydroferulic, DHFA*)	0.8 ± 0.3	0.08 ± 0.07	0.03 ± 0.02	0.07 ± 0.05	0.09 ± 0.04	0.07 ± 0.04	**1.1 ± 0.4**
3-(3′-Hydroxy-4′-methoxyphenyl) propanoic acid (*Dihydroisoferulic, DHiFA*)	N.D.	0.32 ± 0.07	0.38 ± 0.08	1.0 ± 0.2	0.7 ± 0.2	1.0 ± 0.2	**3.2 ± 0.2**
3-(4′-Hydroxyphenyl)propanoic acid (*Dihydrocoumaric acid, DHCoA*)	2.4 ± 0.8**	0.4 ± 0.2**	0.3 ± 0.1*	0.9 ± 0.3	0.8 ± 0.3	1.0 ± 0.2	**6 ± 2****
3-(3′,4′-Dimethoxyphenyl)propanoic acid (*Dihydrodimethoxycinnamic acid*)	N.D.	0.08 ± 0.01	0.06 ± 0.01	0.12 ± 0.03	0.12 ± 0.04	0.25 ± 0.05	**0.63 ± 0.08**
3-(4′-Hydroxyphenyl)propanoic acid-3′-glucuronide (*DHCA-3′-glucuronide*)	N.D.	0.11 ± 0.04	0.06 ± 0.02	0.08 ± 0.02	0.007 ± 0.03	0.20 ± 0.06*	**0.5 ± 0.1**
3-(3′-Hydroxyphenyl)propanoic acid-4′-sulfate (*DHCA-4′-sulfate*)	7 ± 2**	0.6 ± 0.2*	0.20 ± 0.04	0.5 ± 0.1	0.6 ± 0.2	1.7 ± 0.5*	**10 ± 2***
3-(4′-Hydroxyphenyl)propanoic acid-3′-sulfate (*DHCA-3′-sulfate*)	N.D.	0.7 ± 0.2	0.6 ± 0.2	2.5 ± 0.6	3 ± 1	2 ± 1*	**9 ± 3**
3-(3′-Methoxyphenyl)propanoic acid-4′-glucuronide (*DHFA- 4′-glucuronide*)	3 ± 1**	0.21 ± 0.06	0.34 ± 0.09	1.0 ± 0.2	0.8 ± 0.2	1.0 ± 0.3	**7 ± 1***
3-(4′-Methoxyphenyl)propanoic acid-3′-glucuronide (*DHiFA- 3′-glucuronide*)	N.D.	0.3 ± 0.1	0.28 ± 0.07	1.1 ± 0.2	0.8 ± 0.4	0.6 ± 0.3	**3.1 ± 0.7**
3-(3′-Methoxyphenyl)propanoic acid-4′-sulfate (*DHFA- 4′-sulfate*)	N.D.	0.6 ± 0.1	0.8 ± 0.2	2 ± 1	3 ± 1	2.6 ± 0.8	**9 ± 2**
3-(4′-Methoxyphenyl)propanoic acid-3′-sulfate (*DHiFA- 3′-sulfate*)	N.D.	0.14 ± 0.04	0.09 ± 0.03	0.24 ± 0.09	0.11 ± 0.03	2.0 ± 0.7	**2.6 ± 0.8**
3-(Phenyl)propanoic acid-4′-glucuronide (*DHCoA-4′-glucuronide*)	N.D.	0.23 ± 0.09*	0.16 ± 0.05**	0.56 ± 0.2*	0.4 ± 0.1*	1.0 ± 0.1**	**1.9 ± 0.3**
3-(Phenyl)propanoic acid-4′-sulfate (*DHCoA-4′-sulfate*)	N.D.	2.0 ± 0.8*	1.5 ± 0.5	5.0 ± 2	5 ± 2	7 ± 3*	**21 ± 4**
3-Dihydrocaffeoylquinic acid	N.D.	0.025 ± 0.007	0.019±0.005*	0.04 ± 0.01	0.03 ± 0.01	0.15 ± 0.06*	**0.27 ± 0.08****
5-Dihydrocaffeoylquinic acid	N.D.	0.004 ± 0.003	0.002 ± 0.001	0.002 ± 0.002	0.002 ± 0.002	0.015 ± 0.008	**0.03 ± 0.01**
4-Dihydrocaffeoylquinic acid	N.D.	0.008 ± 0.004	0.003 ± 0.002	0.004 ± 0.002	0.004 ± 0.003	0.02 ± 0.01	**0.04 ± 0.02**
3-Dihydroferuloylquinic acid	N.D.	0.11 ± 0.02*	0.062±0.008*	0.08 ± 0.01**	0.08 ± 0.02**	0.14 ± 0.02	**0.5 ± 0.07**
5-Dihydroferuloylquinic acid	N.D.	0.08 ± 0.04	0.021±0.008*	0.03 ± 0.02	0.008 ± 0.003	0.04 ± 0.02	**0.18 ± 0.07**
4-Dihydroferuloylquinic acid	N.D.	0.015 ± 0.008	0.007 ± 0.004	0.011 ± 0.007	0.003 ± 0.002	0.009 ± 0.005	**0.05 ± 0.02**
Dihydrocoumaroylquinic acid	N.D.	0.03 ± 0.02	0.009 ± 0.007	0.09 ± 0.06	0.17 ± 0.06	0.09 ± 0.03*	**0.4 ± 0.1**

Dihydrocoumaroylquinic acid	N.D.	0.017 ± 0.007	0.010 ± 0.003	0.015 ± 0.003	0.014 ± 0.005	0.12 ± 0.06*	**0.17 ± 0.07***
Feruloylglycine	14 ± 4*	1.0 ± 0.3	1.2 ± 0.3	3 ± 1	3 ± 1	3 ± 1	**25 ± 5**
*Iso*Feruloylglicine	N.D.	0.13 ± 0.05**	0.10 ± 0.02**	0.12 ± 0.04	0.06 ± 0.02*	0.17 ± 0.05	**0.6 ± 0.1**
**TOTAL – Colonic absorption**	**42 ± 10****	**8 ± 2**	**8 ± 2**	**23 ± 4**	**21 ± 6**	**30 ± 8**	**132 ± 17**
**Other microbial metabolites**							
3′,4′-Dihydroxyphenylacetic acid	0.6 ± 0.1*	0.15 ± 0.03	0.05 ± 0.02	0.08 ± 0.02	0.09 ± 0.03*	0.31 ± 0.03	**1.3 ± 0.2***
4′-Hydroxy-3′-methoxyphenylacetic acid	5.6 ± 0.8	1.5 ± 0.2	0.8 ± 0.1	1.0 ± 0.2	0.68 ± 0.15	2.9 ± 0.7*	**12 ± 1**
3′-Hydroxyphenylacetic acid	4 ± 1	1.0 ± 0.2	0.7 ± 0.2	0.7 ± 0.1	0.6 ± 0.2	1.8 ± 0.6*	**9 ± 2**
3,4-Dihydroxybenzoic acid	0.06 ± 0.03	0.03 ± 0.01*	0.014 ± 0.003	0.027 ± 0.007	0.018 ± 0.007	0.030 ± 0.009	**0.18 ± 0.04**
4-Hydroxybenzoic acid	0.34 ± 0.06*	0.14 ± 0.02	0.10 ± 0.01	0.09 ± 0.02	0.08 ± 0.02	0.26 ± 0.03	**1.01 ± 0.09***
3-Hydroxybenzoic acid	0.47 ± 0.08	0.10 ± 0.01	0.06 ± 0.01	0.11 ± 0.02	0.07 ± 0.02	0.19 ± 0.04*	**1.0 ± 0.1**
4′-Hydroxyhippuric acid	7 ± 1	1.5 ± 0.3	0.6 ± 0.1	1.2 ± 0.2	1.1 ± 0.3	2.8 ± 0.6	**14 ± 2**
3′-Hydroxyhippuric acid	18 ± 5**	4 ± 1*	2.6 ± 0.7**	6 ± 2	5 ± 1	9 ± 2*	**46 ± 8****
**TOTAL – Other microbial metabolites**	**36 ± 5**	**9 ± 2**	**5.0 ± 0.8**	**9 ± 2**	**8 ± 2**	**18 ± 3***	**85 ± 8**
**TOTAL Colonic + other microbial met.**	**79 ± 14****	**17 ± 3**	**13 ± 2***	**32 ± 6**	**29 ± 7**	**48 ± 10**	**217 ± 21**
**TOTAL INTESTINAL+COLONIC+OTHERS**	**79 ± 14****	**28 ± 5**	**19 ± 3**	**44 ± 6**	**39 ± 10**	**61 ± 15**	**274 ± 30**

Values represent mean ± standard error (n=9). *$p < 0.05$ **$p < 0.01$ week-0 vs. week-8. N.D.: Not detected.
